# Subpopulations in clinical samples of *M. tuberculosis* can give rise to rifampicin resistance and shed light on how resistance is acquired

**DOI:** 10.1093/jacamr/dlaf175

**Published:** 2025-10-09

**Authors:** Viktoria M Brunner, Philip W Fowler

**Affiliations:** Nuffield Department of Medicine, University of Oxford, Oxford, UK; Nuffield Department of Medicine, University of Oxford, Oxford, UK; National Institute of Health Research Oxford Biomedical Research Centre, John Radcliffe Hospital, Oxford, UK; Health Protection Research Unit in Healthcare Associated Infections and Antimicrobial Resistance, University of Oxford, Oxford, UK

## Abstract

**Objectives:**

WGS has become a key tool for diagnosing *Mycobacterium tuberculosis* infections, but discrepancies between genotypic and phenotypic drug susceptibility testing can hinder effective treatment and surveillance. This study investigated the impact of resistant subpopulations and compensatory mutations in WGS-based rifampicin resistance prediction.

**Methods:**

Based on a dataset of 35 538 clinical *M. tuberculosis* samples, the sensitivity and specificity of resistance classification were evaluated with and without considering subpopulations and compensatory mutations.

**Results:**

By lowering the fraction of reads required to identify a resistance-associated variant in a sample from 0.90 to 0.05, the sensitivity increased significantly from 94.3% to 96.4% without a significant impact on specificity. Allowing compensatory mutations to predict resistance further lowered the false negative rate. Finally, we found that samples with resistant subpopulations were less likely to be compensated than homogeneous resistant samples. Further analysis of these samples revealed distinct clusters with differing amounts of within-sample diversity, pointing towards different mechanisms of resistance acquisition, such as within-host evolution and secondary infections.

**Conclusions:**

Our results indicate that a substantial fraction of false negative calls in WGS-based rifampicin resistance prediction can be explained by masked resistant subpopulations. The genetic diversity within the heterogeneous samples is consistent with at least 28% of the rifampicin resistance arising from secondary infections.

## Introduction

TB is estimated to be responsible for 1.25 million deaths in 2023, with 10.8 million people developing disease.^1^ Resistance to antibiotics is particularly concerning, with MDR-TB estimated to cause 150 000 deaths that year despite only 400 000 people developing MDR-TB.^1^ Hence, the first step towards successfully treating *Mycobacterium tuberculosis* infections is fast and reliable diagnostics, including drug susceptibility testing (DST). Phenotypic DST involves culturing the bacteria in the presence of different antibiotics,^2^ and is reliable for most drugs. However, since *M. tuberculosis* grows slowly, it is time consuming despite efforts to reduce culture time.^3^ Genotypic DST instead uses the presence of genetic variants to infer whether a sample is resistant and can be faster than phenotypic DST.^4^ The prerequisites are that the underlying mechanism is genetic and the exact genetic variants conferring resistance are known. If true, one can then look for the presence of specific genetic variation using rapid molecular tests such as GeneXpert MTB/RIF,^5^ or use WGS to scan the entire bacterial genome for resistance-associated variants (RAVs) for different antibiotics.^6^ The latter requires WGS of the patient-derived sample, followed by application of a high-confidence catalogue of RAVs, enabling the sample to be classified as susceptible or resistant to a panel of antibiotics.^7^ Due to its cost and complexity, WGS will not become a front-line diagnostic method in many high-burden countries in the near future but could fill the need for ongoing national surveillance to identify resistant strains in circulation not detected by cheaper molecular tests.^8^

The second edition of the WHO catalogue of mutations contains the most comprehensive list of RAVs to date for predicting rifampicin resistance in *M. tuberculosis* and achieves 93.3% sensitivity and 96.9% specificity on its training dataset compared with standard phenotypic DST results.^9^ Despite rifampicin being one of the best-performing drugs, the sensitivity for rifampicin resistance prediction remains below the 95% threshold proposed for antimicrobial susceptibility test devices by the International Standards Organization (ISO).^10^ It is, of course, unrealistic to expect perfect agreement between the results of phenotypic and genotypic DST, but any discrepancy creates problems not only for scientific efforts investigating disease transmission networks of *M. tuberculosis*,^11^ but also for diagnostics.^12^ If WGS-based DST is to complement or even replace phenotypic DST in some settings, it needs to achieve comparable performance. Hence there is a strong need to close the gap between phenotypic and genotypic DST results.

Here, we show that the performance of WGS-based DST for rifampicin can be trivially improved by permitting resistant subpopulations to contribute to the final classification. This has already been shown to significantly improve the sensitivity of resistance prediction for fluoroquinolones in *M. tuberculosis*.^13^ The hypothesis is that the genotypic approach is under-calling resistance since resistant subpopulations are not identified by many WGS bioinformatics pipelines, yet phenotypic DST methods will flag samples as resistant if as few as 1% of the bacteria are resistant.^14^ The phenomenon of heteroresistance has been described in many pathogens.^15^ However, for most pathogens, resistant subpopulations are not expected since the standard laboratory process is to pick single colonies, constraining the detection of any heteroresistance. This is not true for *M. tuberculosis* where e.g. an aliquot taken for DNA extraction from a BD Mycobacteria Growth Indicator Tube (MGIT^™^) tube usually contains multiple ‘crumbs’^16^ and so could readily harbour resistant subpopulations, reflecting more accurately the patient’s infection.

We define the fraction of read support (FRS) as the proportion of reads at a genetic locus that supports a specific genetic variant. Bioinformatics pipelines conventionally specify a minimum FRS for a genetic variant to be called. The first edition of the WHO *M. tuberculosis* mutation catalogue^17^ and the CRyPTIC consortium^18^ both used a conservative FRS threshold of 0.90 when calling genetic variants. This high threshold prevents sequencing errors leading to spurious variant calls, but as sequencing technologies have improved and error rates decreased, this now seems unduly conservative since it also ensures genetic subpopulations are not detected. Such subpopulations are expected if an infection has only recently evolved resistance, perhaps in response to treatment, or if there has been a secondary resistant infection.

A second way to improve the performance of catalogue-based predictions is to include more RAVs. For now, catalogues only contain alleles that (are assumed to) directly cause resistance. We show that including genetic variants indirectly linked to resistance, such as compensatory mutations (CMs), can improve sensitivity without lowering specificity. CMs arise in response to a reduction in fitness caused by rifampicin resistance mutations and hence although not causal are only found in rifampicin-resistant samples.^19–21^ Hence including them in a catalogue would mainly be useful for samples where the RAV cannot be resolved, such as those with low sequencing quality. We have previously identified a list of high-confidence CMs using their strong association with the presence of resistance mutations.^22^ We aimed to test if including this list in the official WHO resistance catalogue^9^ improved the performance metrics of resistance prediction.

**Figure 1.**
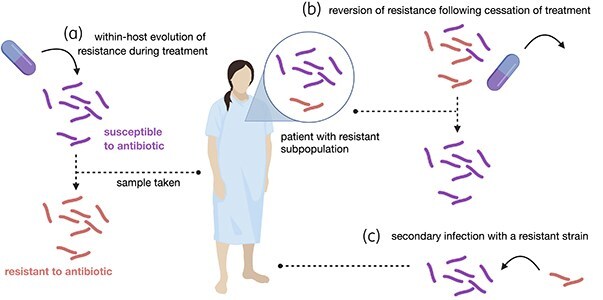
Three ways a patient can acquire an infection with a resistant subpopulation. Samples with resistant subpopulations can originate from multiple different scenarios, among them (a) within-host evolution, (b) reversion to susceptible phenotype following cessation of treatment, and (c) secondary infection.

Samples with resistant subpopulations capture an interesting state in *M. tuberculosis* infections that can arise via multiple different scenarios (Figure 1). The first possibility is within-host evolution of resistance, where the resistant subpopulation gradually takes over a susceptible population during drug treatment (Figure 1a). Secondly, the evolved drug-resistant subpopulation can revert to a susceptible phenotype or be outcompeted after drug treatment is stopped (Figure 1b); or thirdly the resistant subpopulation is acquired through a secondary infection (Figure 1c). Distinguishing between these possibilities requires that we know whether the sample was taken before, during or after drug treatment. Unfortunately, precise data on this are scarce in WGS datasets and hence we can seldom distinguish between scenarios 1 and 2 (Figure 1a and b). The second unknown variable is the source of resistance: within-host evolution or a secondary infection. We aimed to discern the source of resistance by quantifying the amount of genetic diversity in the samples with resistant subpopulations in our dataset.

## Materials and methods

### Dataset sources

In addition to collecting *>*20 000 *M. tuberculosis* samples, each of which underwent WGS and DST using one of two bespoke 96-well broth microdilution plates,^18,23^ the Comprehensive Resistance Prediction for Tuberculosis: an International Consortium (CRyPTIC) project also aggregated *M. tuberculosis* samples with WGS and/or DST data that had been previously published. An earlier version of this dataset with heterogeneous DST methods was used to build the first edition of the WHO catalogue of TB RAVs.^24^

### WGS data processing

A subset of 41 575 publicly available samples from the European Nucleotide Archive with short-read paired-end FASTQ files (version 3.0.0 of the CRyPTIC dataset^25^) were uploaded to the EIT Global Pathogen Analysis Service (GPAS; https://gpas.global) and processed using version d5f9cd0 of the Mycobacterial pipeline.^26^ The variant caller, Clockwork, calculates the FRS and, by default, only calls genetic variants where FRS ≥0.90 with a filter applied to the remainder.^27^ Here, we instructed the downstream tool, gnomonicus, to ignore these filters and record all potential genetic variants so that we could detect subpopulations of bacteria.^28^ Most samples (37 594) had one or more of 101 020 mutations detected in the RNA polymerase (genes *rpoA*, *rpoB*, *rpoC*, *rpoZ* and *sigA*). These include 3260 so-called null mutations where there are insufficient (*<*3) reads to call a variant. To protect against spurious calls due to sequencing errors, variants needed to be supported by at least three reads.

Examining *rpoB* in more detail we found 31 347 samples containing 58 789 mutations with an FRS ≥ 0.90 and 1372 samples containing 1940 mutations supported by an FRS *<* 0.90. A small number of the latter samples had very large numbers of putative mutations, indicative of contamination or poor sequencing quality. In both cases the most common *rpoB* mutations were A1075A and S450L, as expected. Mutations were flagged as being associated with resistance to rifampicin according to the second edition of the WHO catalogue of mutations in *M. tuberculosis*.^9^ We used a published list of high-confidence CMs^22^ to annotate which mutations are compensatory.

### Phenotypic DST data processing

A total of 52 148 samples had one or more binary rifampicin DST results using a range of methods; the most common were broth microdilution plate (24 172 samples) and mycobacterial growth indicator tube (23 682). If a sample had been tested using more than one method and all methods produced the same Susceptible/Resistant result then, if present, the CRyPTIC result was retained since it had richer data and had undergone additional quality control. If the methods disagreed on the outcome then the first resistant result was retained. This resulted in 48 031 samples with a single rifampicin DST result. Merging with the genetic data identified 35 538 samples that had both WGS data and a single rifampicin DST binary result: this dataset formed the basis of our subsequent analysis. The sensitivity and specificity of the genotypic resistance call were calculated with respect to the phenotypic drug susceptibility result. Significance was tested using a proportions Z-test.

### Reproducibility statement

All analysis, figures and tables in this article can be reproduced using a GitHub repository that contains all data tables and code.^29^

## Results

### Classifying subpopulations that contain rifampicin RAVs as resistant improves sensitivity

**Figure 2.**
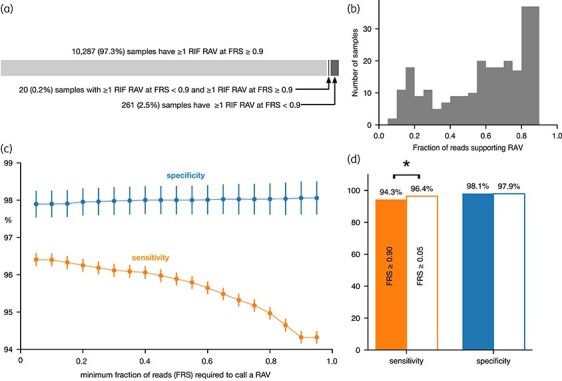
Lowering the fraction of read support (FRS) threshold for calling rifampicin (RIF) resistance-associated variants (RAVs) increases sensitivity with no significant effect on specificity. (a) The majority of samples containing a RAV are homogeneous, meaning they show a RIF RAV at ≥0.90 FRS. The heterogeneous samples make up 2.5% of samples, but we rarely see samples with both a RAV at ≥0.90 FRS and a RAV with lower read support. (b) The distribution of FRS for the RAVs in the 258 heterogeneous samples with 0.05 ≤ FRS *<* 0.90. (c) Decreasing the FRS threshold required to support a variant call that is a known RAV increases sensitivity of the prediction with little effect on specificity. The slopes of a linear regression for sensitivity and specificity are −0.02 and 0.002, respectively. Error bars (95% confidence limits) are plotted as calculated via the binomial proportion. (d) Sensitivity is significantly improved if the FRS threshold is lowered from 0.90 to 0.05 (Z-test, *P* value = 8 ×10^−13^). There is no significant change in specificity. For clarity error bars are not plotted.

In our dataset of 35 538 samples, we describe samples with a rifampicin RAV with an FRS ≥ 0.90 as *homogeneous*, and samples containing RAVs at an FRS *<* 0.90 as *heterogeneous*. Any mutation (including an RAV) with an FRS *<* 0.90 is described as *minor*. Of the 10 568 samples containing a rifampicin RAV at any level of read support, 10 287 were homogeneous, 261 were heterogeneous and 20 were mixed, i.e. containing at least two rifampicin RAVs, one supported by an FRS ≥ 0.90 and another with an FRS *<* 0.90 (Figure 2a). Minor rifampicin RAVs were seen down to an FRS of 0.085, possibly increasing at higher values of FRS (Figure 2b). To detect a minor RAV at an FRS of 0.05 necessitates a read depth of 60 since three reads are required to call a minor RAV to avoid false positives; this was satisfied, on average, for 79.8% of the dataset (Figure S1, available as Supplementary data at *JAC-AMR* Online). This indicates that the full range of values should be considered when assessing the impact of the FRS threshold on the performance of resistance prediction.

The sensitivity increased from 94.3% to 96.4% as the minimum FRS required to call a RAV was decreased from 0.90 to 0.05; over the same range the specificity fell slightly from 98.1% to 97.9% (Figure 2c, Table 1). The increase in sensitivity when the FRS is dropped from 0.90 to 0.05 is statistically significant, whereas the decrease in specificity is not (Figure 2d). Over the same range the negative predictive value (NPV) also significantly increased whereas there was no significant change in the positive predictive value (PPV; Figure S2, Table 1). Taken together, this indicates that applying a conservative FRS threshold masks resistant subpopulations, resulting in falsely predicting some samples as susceptible to rifampicin.

**Table 1.** Fraction of read support (FRS) and corresponding contingency table values and performance metrics for different scenarios of the catalogue-based predictions for rifampicin resistance^a^

RAVs	CMs	*rpoC* F452L	FRS_min_	TP	FP	TN	FN	Sen.	Spec.	PPV	NPV
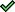		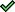	0.90	9819	488	24 640	591	94.3%	98.1%	95.3%	97.7%
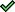		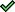	0.05	10 036	529	24 599	374	96.4%	97.9%	95.0%	98.5%
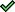	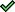	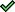	0.90	9842	490	24 638	568	94.5%	98.0%	95.3%	97.7%
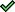	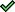	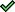	0.05	10 047	663	24 465	363	96.5%	97.4%	93.8%	98.5%
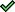	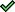		0.05	10 047	531	24 597	363	96.5%	97.9%	95.0%	98.5%
	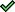	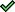	0.05	4452	225	24 903	5958	42.8%	99.1%	95.2%	80.7%

CM, compensatory mutation; FN, false nagative; FP, false positive; 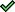, included in scenario; FRS_min_, minimum fraction of reads at a genetic locus needed to call a variant; NPV, negative predictive value; PPV, positive predictive value; RAV, resistance-associated variant; TN, true negative; TP, true positive.

^a^Scenarios are shown for different FRS thresholds, and are either using RAVs and/or CMs for prediction or not. In one scenario, the CM *rpoC* F452L was removed from the analysis. ‘Sen.’ shows sensitivity and ‘Spec.’ the specificity of the resistance prediction.

### CMs can identify rifampicin resistance by association with high specificity

Even when a low FRS threshold was set (e.g. 0.05) we still observed false negatives in our genotypic resistance prediction and this is unlikely to improve much upon further lowering of the FRS threshold (Figure 2c). Another cause for false negative calls is low coverage or sequencing errors in genomic regions associated with resistance; hence it would be useful if there was redundancy when predicting resistance using genetic features. We have previously identified 51 CMs,^22^ which are by definition only present in resistant samples. Hence by allowing samples to be predicted as rifampicin-resistant if they contain either a RAV and/or a CM one might expect to reduce the false negative rate. To test this, we appended these 51 CMs to the WHO catalogue of RAVs for rifampicin.

Including CMs reduced the number of false negative calls from 591 to 568 when applying an FRS threshold of 0.90 to call variants (Table 1), corresponding to a 3.9% decrease. The resulting improvement in sensitivity was not significant, probably due to the small number of affected samples compared with the size of the dataset. Repeating the experiment setting the FRS threshold to 0.05 increased the number of samples falsely predicted to be resistant from 529 to 663. Most (99%) of these erroneous calls were due to samples that despite containing the putative CM *rpoC* F452L were not actually resistant. Excluding this CM *post hoc* did not decrease the specificity when allowing CMs to predict resistance at low FRS (Table 1).

To evaluate how well CMs identify rifampicin resistance on their own, we checked the specificity of using only CMs to predict the resistance phenotype. As expected, only moderate sensitivity was achieved (42.8%, Table 1), since only a fraction of resistant samples exhibit compensation. Interestingly, the specificity of predicting resistance was higher (99.1%) than when simply applying the WHOv2 catalogue of mutations (Table 1). Overall this suggests that permitting CMs to trigger a resistance call could be useful in samples where the RAV has not been resolved, for example if the coverage or read depth is poor, but that we should not rely on them exclusively.

The combined effect of lowering the FRS threshold to 0.05 and permitting CMs to predict resistance was a reduction of false negative calls by 38.6%. Hence more than one-third of the resistant samples incorrectly classified as susceptible were thereby explained and corrected (Table 1), a handy improvement.

### Heterogeneous resistant samples are less likely to show compensation

**Figure 3.**
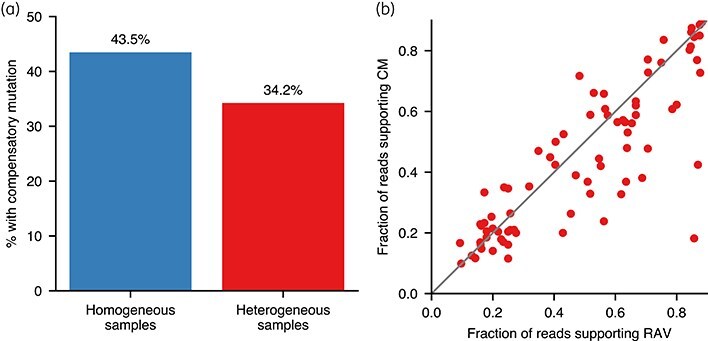
Heterogeneous resistant samples are less likely to also have a compensatory mutation (CM) than homogeneous resistant samples. (a) Percentage of samples showing CMs at any fraction of read support (FRS) in homogeneous versus heterogeneous resistant samples (Fisher’s exact *P* = 3.63 ×10^−4^). (b) For the heterogeneous samples that are compensated, this plot shows the significant correlation between the FRS of the respective resistance and compensatory mutation (*P* = 1.69 ×10^−22^). The grey line indicates the hypothetical line of perfect correlation.

The proportion of heterogeneous samples with a CM was significantly lower than the fraction of homogeneous samples that are compensated (43.5% versus 34.2%; Fisher’s exact test, *P* value = 3.63 × 10^−4^; Figure 3a). Since the fraction of reads supporting the CM and RAV is correlated in the compensated heterogeneous samples (Figure 3b, *P* = 1.69 × 10^−22^) it is likely that they are composed of a susceptible strain and a rifampicin-resistant strain that has acquired a CM.

A smaller proportion of resistant heterogeneous samples contained an *rpoB* S450L mutation: 60.5% compared with 68.6% in homogeneous samples. Since most CMs (96%) co-occur with this mutation, this effect would lead to the proportion of heterogeneous samples with a CM being reduced by 4.9% points to 38.6%. This is 4.4% points higher than our observed fraction (34.2%) and therefore we can explain around half the difference. One possible explanation for the remainder is resistant subpopulations evolving, on average, resistance more recently than homogeneous resistant samples.

### At least 28% of heterogeneous resistant samples are the result of secondary infections

We can gain some insight into the source of resistance by examining genetic diversity in the heterogeneous samples. If the resistant subpopulation arose by within-host evolution (Figure 1a), one would expect to see relatively few, if any, other minor mutations in the sample, given the low mutation rate (0.04–2.2 SNPs per genome per year^30^) of *M. tuberculosis*. A similar logic applies when a sample is taken midway through reversion of resistance following cessation of treatment (Figure 1b); again, relatively few other minor mutations would be expected. If the patient has been infected more than once (Figure 1c), the sample will probably contain different strains and therefore we would expect a much greater number of minor mutations. This will not necessarily always be true: if the coinfecting strain is part of the same outbreak, the number of minor mutations will again be small. Only when the number of minor mutations is high, e.g. if the sample contains multiple (sub)lineages, can we draw a definite conclusion since in this case secondary infection is the only viable scenario.

**Figure 4.**
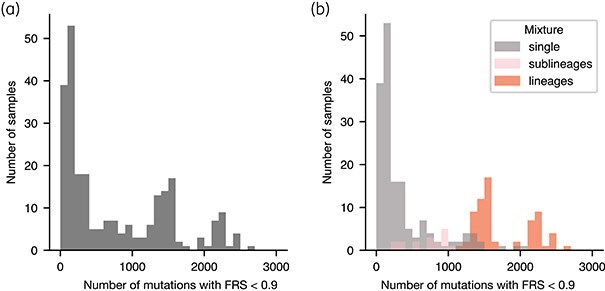
Quantifying genetic diversity within heterogeneous resistant samples using the amount of minor mutations detected. (a) Genetic diversity within the heterogeneous samples with resistant subpopulations, as measured by the amount of minor mutations per sample. Minor mutations are all variants in these samples that display a fraction of read support below 0.90. (b) Same as in (a), but the data are divided into three different batches based on the sample mixture type assigned by mykrobe (v0.12.1): a single lineage, multiple sublineages or multiple lineages per sample. All 85 samples are plotted in Figure S3.

To investigate this, we measured the number of minor mutations in all 248 heterogeneous resistant samples with a singular minor RAV. The latter condition is required to ensure that only a single resistant subpopulation is present. The resulting distribution was very broad; some samples had no or relatively few minor mutations, whereas others had several thousand (Figure 4a).

**Figure 5.**
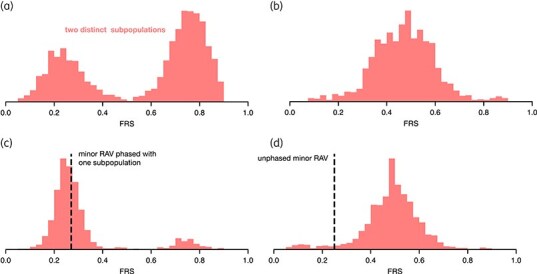
Example fraction of read support (FRS) distributions of minor mutations in heterogeneous samples with multiple (sub)lineages can show if resistance-associated variants (RAVs) are in phase with a subpopulation. (a) FRS distribution in a single sample with multiple (sub)lineages of *M. tuberculosis*. The number of (heterogeneous) mutations at each FRS are shown in histogram representation. The two subpopulations can be clearly distinguished and are around 0.2 and 0.8 in this sample. (b) Plot structure identical to (a), but in this sample we cannot distinguish between the subpopulations due to overlapping FRS counts. (c) Plot structure similar to (a), but additionally a black dashed line indicates the FRS of the RAV in the sample. Here it is in phase with one of the distribution maxima. (d) Plot structure identical to (c), but the dashed line indicating the RAV is out of phase with the distribution maxima, suggesting resistance evolved after transmission.

Mapping the mixture type identified by mykrobe^31^ (single, multiple sublineages, or multiple lineages per sample) onto the distribution neatly segregated the data into three partially overlapping subgroups (Figure 4b). The first was the 163 samples that according to mykrobe consisted of one and only one lineage (labelled ‘single’ in Figure 4b). The remaining 85 samples (34%) were assessed as containing either multiple sublineages belonging to the same lineage, or different lineages. These are labelled ‘sublineages’ and ‘lineages’, respectively, in Figure 4b. The only plausible explanation for the latter two cases is if the patient contracted a secondary infection. To estimate how many of these were resistant at the time of infection we plotted the FRS distribution for all minor mutations present in each of the 85 samples (Figure S3); many samples showed clear bimodality (Figure 5a) whereas others did not (Figure 5b). The latter case is due, we infer, to these samples containing equal parts of all lineages. For 70 of the bimodal samples the FRS of the RAV was in phase with one of the subpopulations (Figures 5c and S3), consistent with the secondary infection being resistant at the point of transmission. The others were either clearly out of phase (Figure 5d) or difficult to call. We conclude that at least 28% (70/248) of the heterogeneous resistant samples were the result of a secondary infection that was resistant at the point of transmission. Interestingly, there was also a significant association between a heterogeneous resistant sample having a CM and showing more than one (sub)lineage (Fisher’s exact test, *P* value = 2.38 × 10^−×^).

## Discussion

We have demonstrated the importance of considering within-host diversity when predicting drug resistance in *M. tuberculosis* using WGS. Although heteroresistance is usually detected by targeted sequencing approaches and rapid molecular tests (e.g. Gene Xpert MTB/RIF), WGS approaches often fail to resolve subpopulations. But by lowering the minimum FRS required to call a RAV from 0.90 to 0.05, we significantly improved the sensitivity of WGS-based genotypic resistance classification by +2.1% to 96.4%, with no detectable effect on specificity. A higher sensitivity improves any treatment decisions and leads to more effective surveillance. Importantly, it also takes rifampicin prediction on this dataset above the required minimum threshold of 95% in both sensitivity and specificity to pass the ISO standard for AST devices.^10^ It would therefore seem sensible for genetic workflows that process *M. tuberculosis* samples to identify and call rifampicin RAVs if their presence is supported by just a few reads, regardless of the FRS. Detecting resistant subpopulations has a greater effect in many second-line drugs. For example, it has been shown that classifying a sample as resistant to moxifloxacin based on a resistant subpopulation improves sensitivity from 85.4% to 94.0%.^13^

Allowing the presence of high-confidence CMs in the RNA polymerase to identify rifampicin resistance by association would also seem a trivial way to boost sensitivity. The improvement in sensitivity on this dataset was not significant, probably due to the moderate read depth (Figure S1) ensuring there were relatively few resistant samples with a CM where the resistance mutations could not be resolved but the CMs could. In scenarios with much lower read depth (e.g. in multiplexed samples) allowing compensatory mutations to predict rifampicin resistance could provide a necessary boost to the sensitivity. In future work we will investigate the read depths at which it becomes beneficial to allow CMs in addition to RAVs to call rifampicin resistance by randomly down-sampling sequencing reads.

As noted above, it is vital that any list of CMs is accurate if such mutations are to be used to infer resistance.^22^ The mutation *rpoC* F452L appears not to be resistance-associated when present at a lower FRS, which could either be an error or point to an inverse causal relationship between this CM and the presence of RAVs. This raises the question of whether F452L should be considered a CM or rather provides an advantageous genetic background for the acquisition of rifampicin resistance, by raising fitness *prior* to resistance emergence. Although speculation, this is supported by the observation that *rpoC* F452L was shown to stabilize the open promoter complex and increase the elongation rate of the *M. tuberculosis* RNA polymerase, not only in the resistant *rpoB* S450L mutant but also in the WT.^32^

Overall, reducing the minimum FRS threshold to 0.05 and permitting the presence of CMs to predict rifampicin resistance raises sensitivity to 96.5%. The remaining discordance can likely be explained by several factors: there may be additional, unknown, rare resistance mutations in the RNA polymerase that have not been classified as such by the second edition of the WHO resistance catalogue. Tackling this problem is non-trivial but could involve the use of machine learning models trained to predict the effect of individual *rpoB* mutations.^33^ There are almost certainly errors in the laboratory processes, such as mislabelling or measurement error, that we can minimize but not eradicate. More straightforwardly, phenotypic methods have been shown to detect 1% heteroresistance,^14^ which is more sensitive than permitted by the mean read depths of this dataset (Figure S1): lowering our threshold from 0.05 to 0.01 would require sequencing to greater depth. Even smaller subpopulations may be relevant; it has been shown that genetics can detect microheteroresistance (0.001 *<* FRS *<* 0.05), and this could be indicative of the subsequent development of phenotypic drug resistance in clinical practice.^34^

We can also gain insight into the timing and origin of resistance to rifampicin by examining the samples with resistant subpopulations. Around half (53%) of the significant reduction in the proportion of compensated heterogeneous resistant samples can be explained by the lower prevalence of the *rpoB* S450L mutation. Explanations for the remaining fraction include resistant heterogeneous samples having acquired resistance more recently and the varying fitness costs of different RAVs. The number of other putative minor mutations in the heterogeneous samples hints at the probable sources of resistance. Our data are consistent with secondary infection, as opposed to within-host evolution, being the root cause of rifampicin resistance in at least 28% of the heterogeneous samples. This is consistent with a longitudinal study that found that of 107 patients with recurrent TB, 54 (50.5%) had become resistant to rifampicin via a secondary infection.^35^ The striking level of genetic diversity detected within our samples is only achievable if the patient is subject to a secondary infection, which likely introduced the resistant subpopulation. The significant association of CMs with samples containing multiple (sub)lineages indicates these samples are also more likely to be compensated. This is consistent with the secondary infection scenario, since the infecting strain could already have been compensated before being transmitted.

Stepping back, it is not surprising that the CRyPTIC dataset contains genetically heterogeneous *M. tuberculosis* samples due to how clinical samples are grown and colonies extracted. For example, aliquots taken from positive MGIT tubes will contain multiple ‘crumbs’ and therefore multiple colonies. This is in sharp contrast to laboratory practices for other pathogens where single colony picks from solid media are commonplace. One should, therefore, perhaps consider mycobacterial genetic sequencing as inherently metagenomic.

In conclusion, this study has shown how to improve the sensitivity of WGS-based rifampicin resistance prediction by including subpopulations, CMs and population diversity. It also provides useful information on how resistance emerges clinically. In general, these findings will facilitate improved diagnostic strategies and more effective management of drug-resistant TB.

## Supplementary Material

dlaf175_Supplementary_Data

## Supplementary data

Figures S1–S3 are available as Supplementary data at *JAC-AMR* Online.
