# Supplementary material for: Subpopulations in clinical samples of M. tuberculosis can give rise to rifampicin resistance and shed light on how resistance is acquired
Source: JAC Antimicrob Resist. 2025 Oct 9;7(5):dlaf175. doi: 10.1093/jacamr/dlaf175 (PMC12509863; doi:10.1093/jacamr/dlaf175)
Supplement: dlaf175_Supplementary_Data [file dlaf175_supplementary_data.pdf]

Subpopulations in clinical samples of *M. tuberculosis* can give rise to rifampicin resistance and shed light on how resistance is acquired – Supplemental Information.

Viktoria M BRUNNER[1] and Philip W FOWLER*[1,2,3]

[1]Nuffield Department of Medicine, University of Oxford, Oxford, UK
[2]National Institute of Health Research Oxford Biomedical Research Centre, John Radcliffe Hospital, Oxford, UK
[3]Health Protection Research Unit in Healthcare Associated Infections and Antimicrobial Resistance, University of Oxford, UK

*To whom correspondence should be addressed: philip.fowler@ndm.ox.ac.uk, @philipwfowler

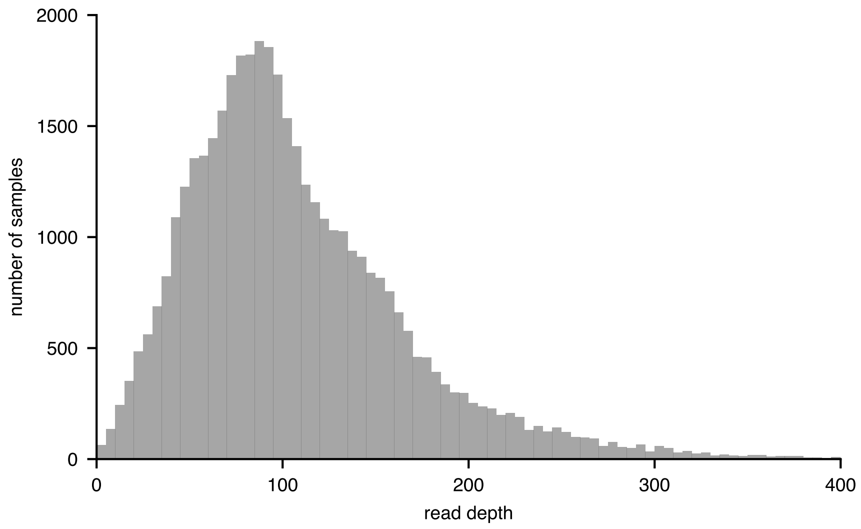

Figure S1: **The samples have a range of mean read depths**. For the 35,538 samples with both WGS and pDST data the mean and modal depth are 108 and 96, respectively. A majority of samples (79.8%) have a mean read depth of at least 60 which permits a minor variant at an FRS of 0.05 to be detected (as three reads are required). The chance of detecting a minor variant at an FRS of 0.01 in this dataset is low because only 1.2% of samples have a mean read depth of 300 or greater.

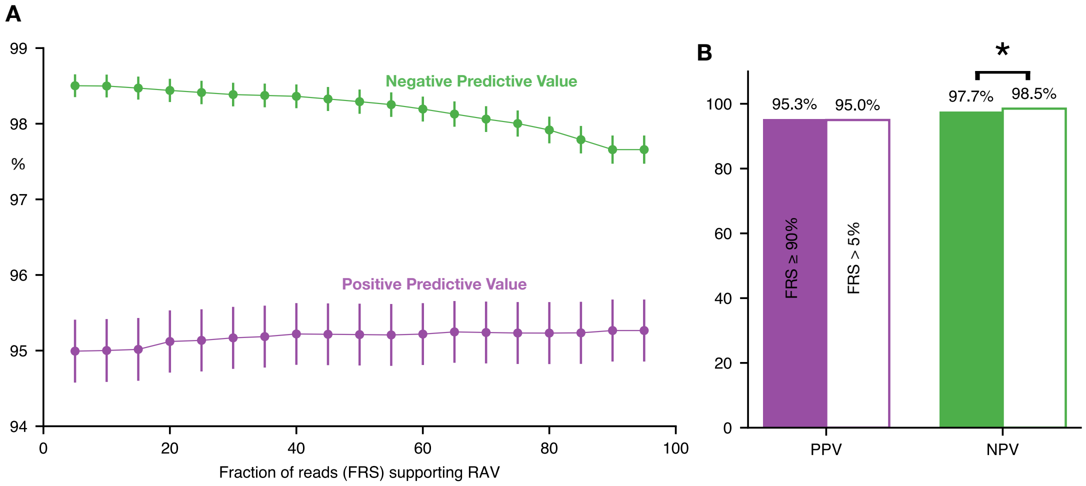

Figure S2: Classifying samples containing a subpopulation with a resistance-associated variant (RAV) as resistant improves the NPV with no effect on PPV. (A) Decreasing the FRS threshold required to support a variant call that is a known RAV increases NPV with little effect on PPV. The slopes of a linear regression for NPV and PPV are -0.009 and 0.003, respectively. Error bars (95% confidence limits) are plotted as calculated via the binomial proportion. **(B)** The NPV is significantly improved if the FRS threshold is lowered from 90% to 5% (z-test, p-value 5e-12). There is no significant difference in the change in PPV. For clarity error bars are not plotted.

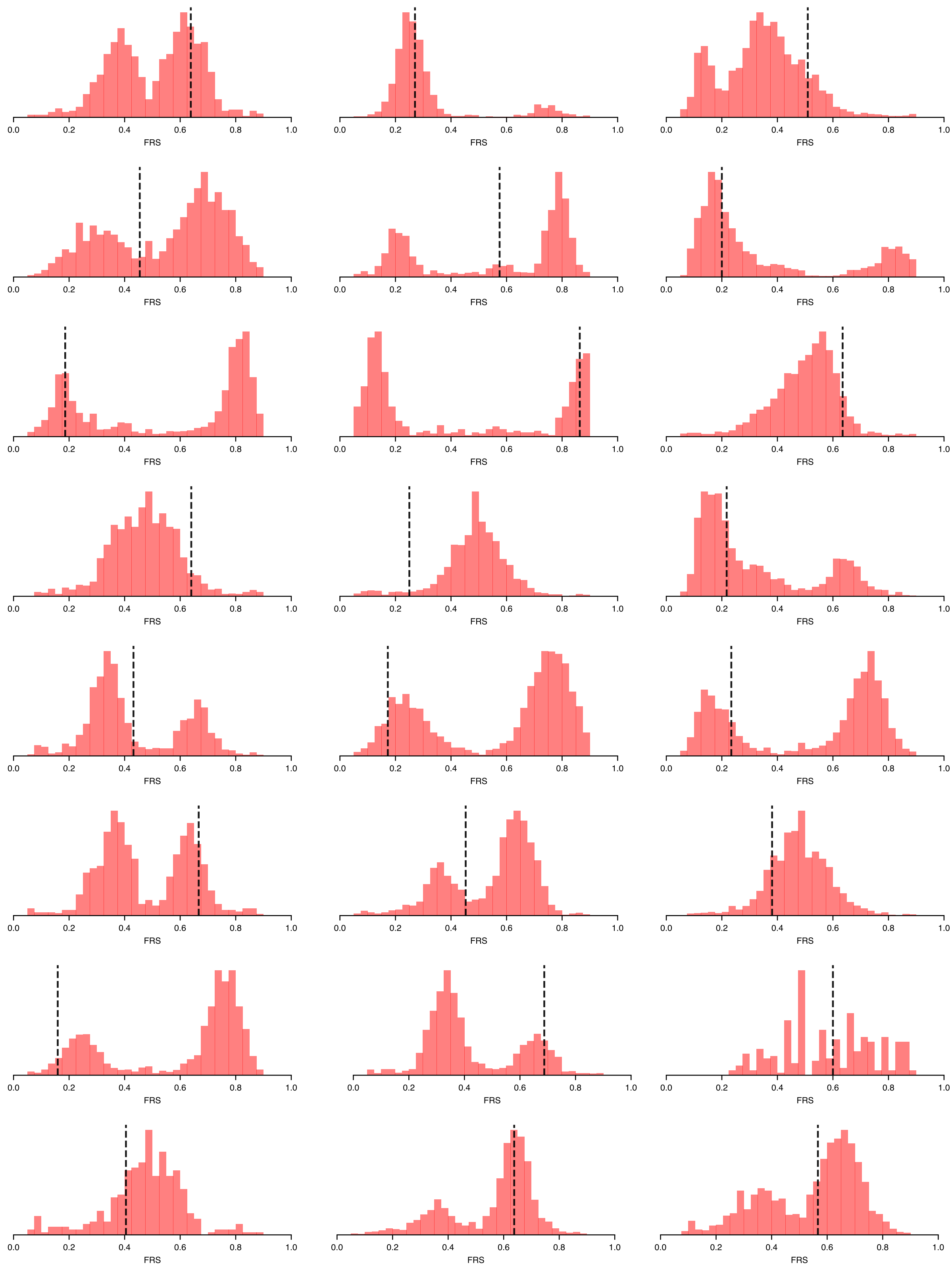

Figure S3: (related to Figure 5) Distributions of the fraction of reads (FRS) supporting all detected minor mutations in the 85 heterogeneous resistant samples that do not have a single lineage (Figure 4). Samples typically show unimodal, bimodal or complex behaviour. The FRS of the detected resistance-associated variant is annotated via a dash black line. If it coincides with one of the subpopulations it is in phase and can be reasonably assumed to not have evolved since transmission. Samples 1-24 (out of 85) shown here.

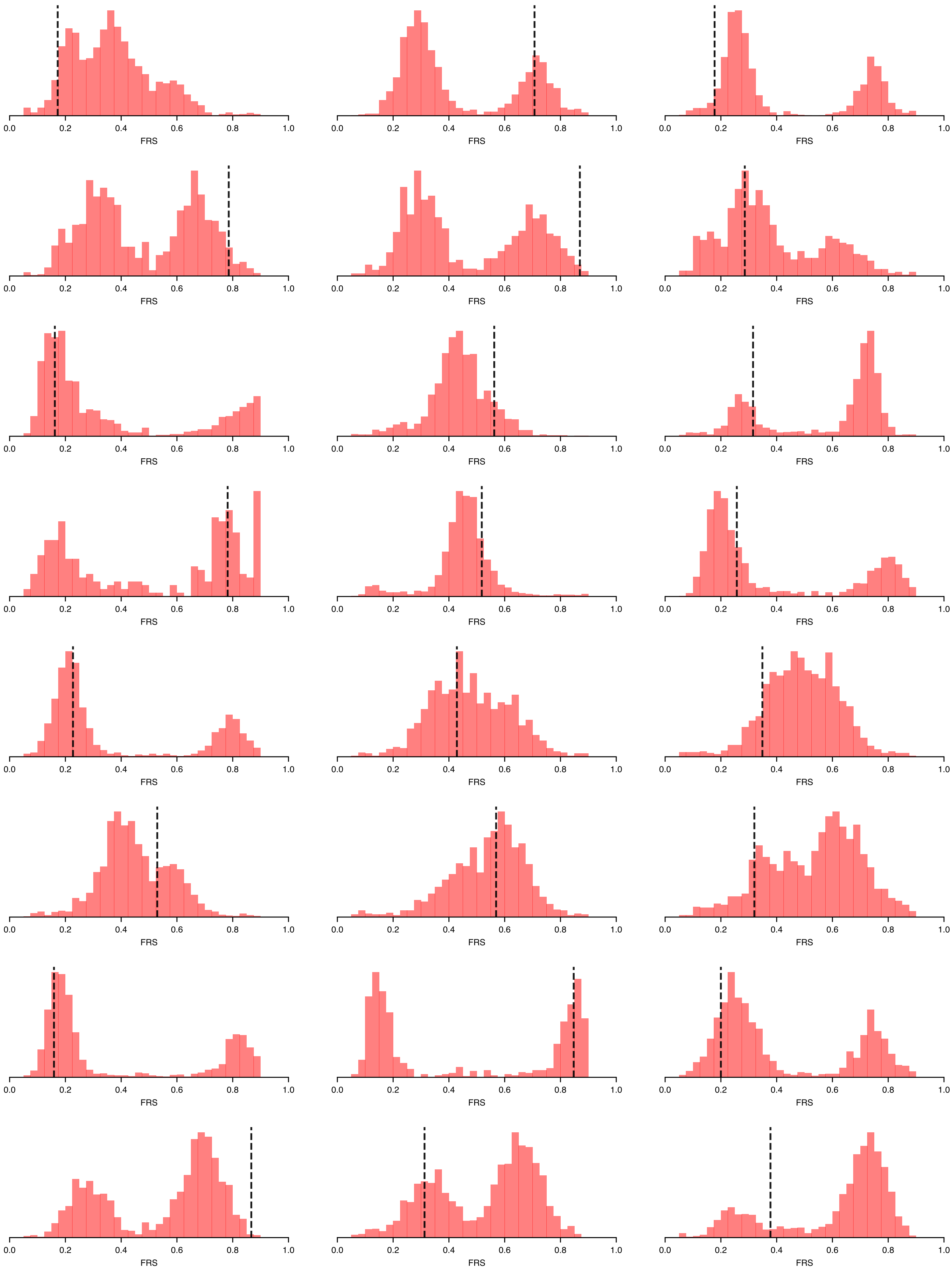

Figure S3 continued: Samples 25-48 (out of 85) shown here.

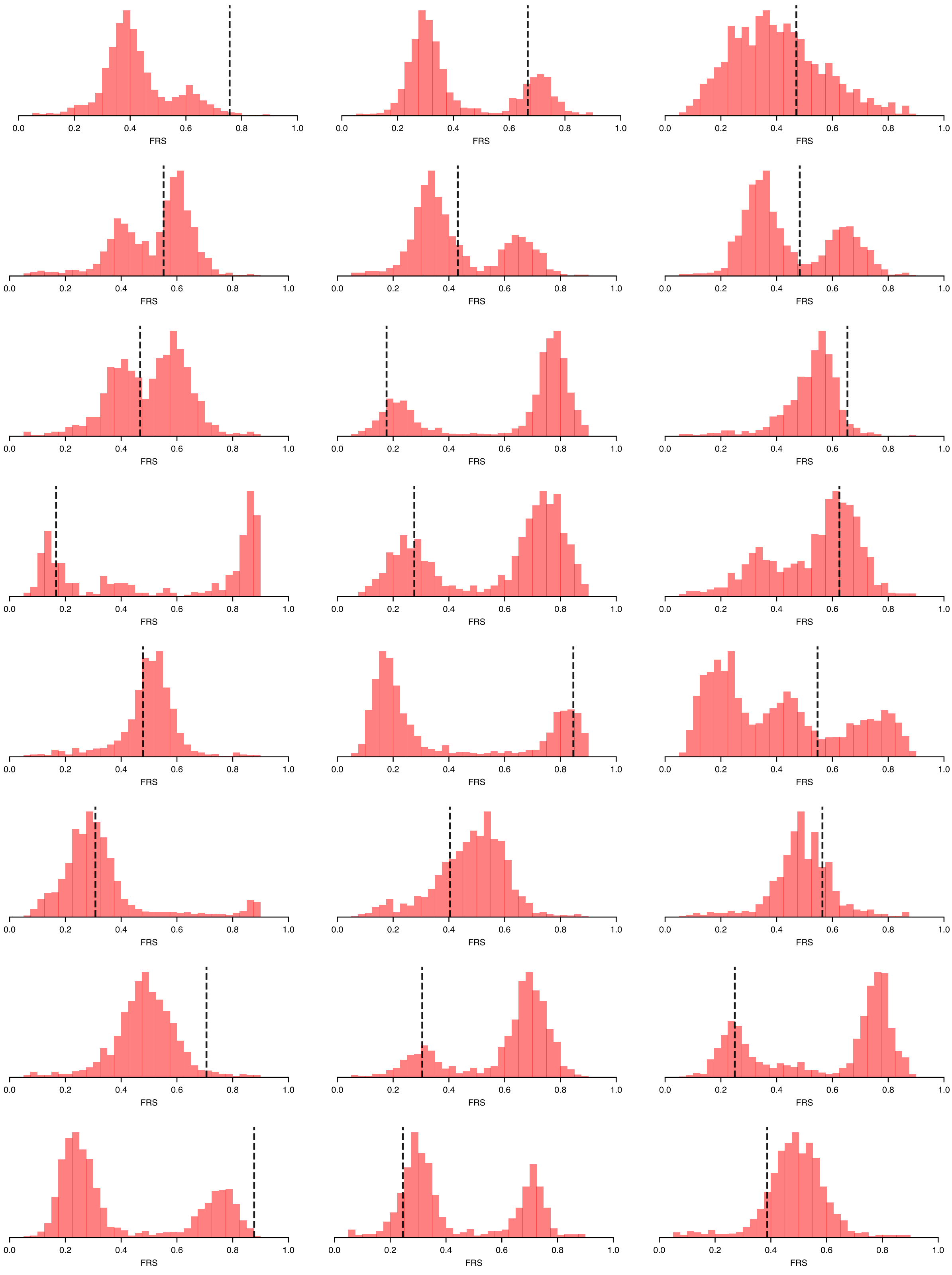

Figure S3 continued: Samples 49-72 (out of 85) shown here.

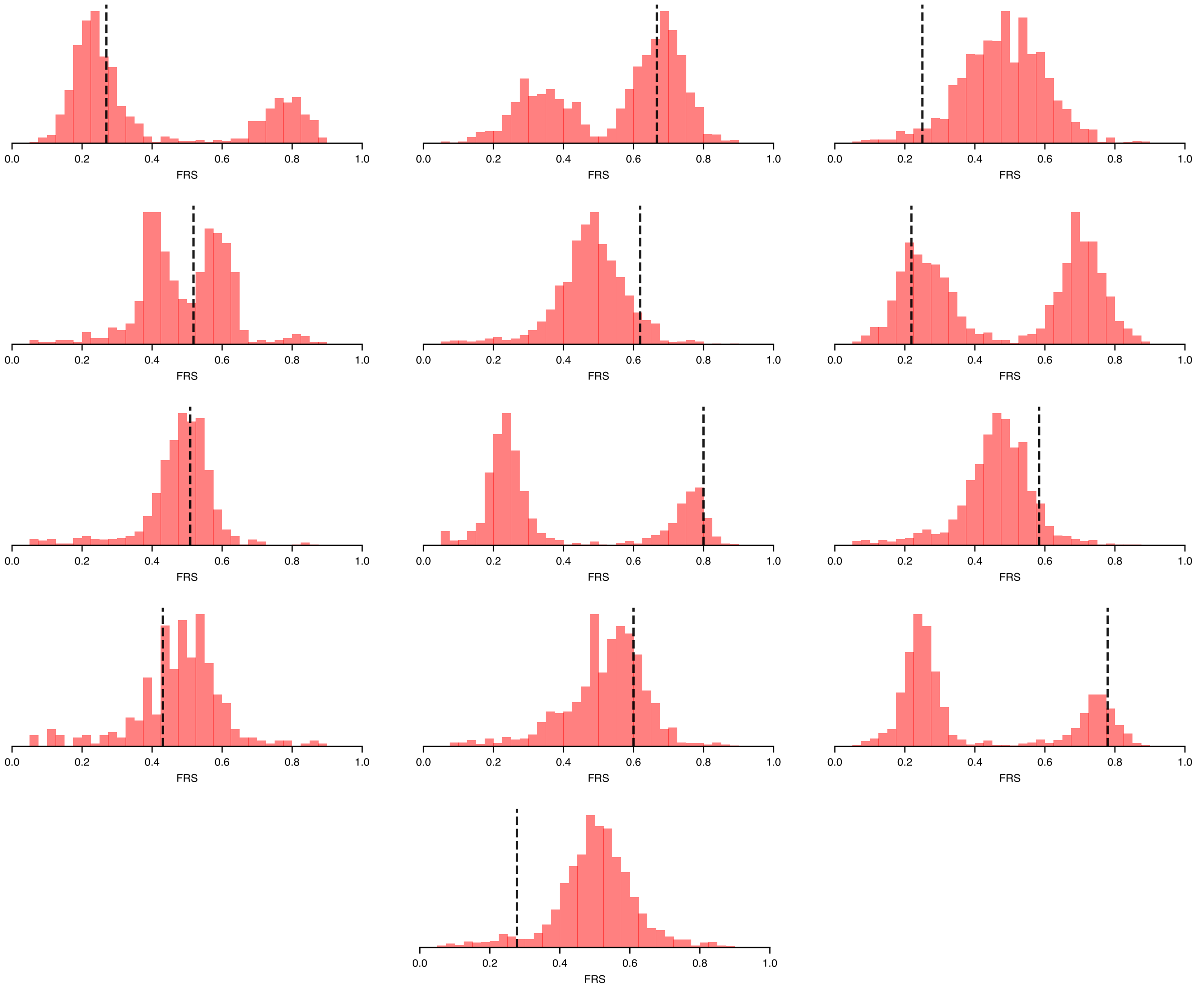

Figure S3 continued: Samples 73-85 (out of 85) shown here.
